# Reopenable clip-over-the-line method for closure of postoperative gastric fistula

**DOI:** 10.1055/a-2615-1542

**Published:** 2025-07-01

**Authors:** Takehide Fukuchi, Shinpei Kondo, Tomo Oka, Shigeru Iwase, Shin Maeda

**Affiliations:** 1 36993 Department of Gastroenterology, Fujisawa City Hospital, Kanagawa, Japan; 2 36993 Department of Acute Care Surgery, Emergency Medical Center, Fujisawa City Hospital, Kanagawa, Japan; 3 26438 Department of Gastroenterology, Yokohama City University Graduate School of Medicine, Yokohama, Japan

Nomura et al. developed the reopenable clip-over-the-line method (ROLM), originally reported for closure of large mucosal defects after endoscopic submucosal dissection (ESD) 1 . Its use has since expanded to managing perforations during ESD and suturing post–full-thickness resections 2 3 . However, no reports have described its use for fistula closure. Common options such as the over-the-scope clip (OTSC) system (Ovesco Endoscopy GmbH) are often applied for fistula closure, but their success rate – especially in the gastric wall – is relatively low 4 . Endoscopic hand-suturing has also emerged as a closure method, though it remains technically complex 5 .

We report the first case of successful gastric fistula closure using ROLM ( Video 1 ). The patient was a 54-year-old woman who developed a postoperative pancreatic fistula (POPF) following distal pancreatectomy for pancreatic cancer ( Fig. 1 **a** ). Despite drainage, the cavity progressively enlarged. On postoperative day (POD) 17, gastrography revealed a gastric fistula connected to the POPF ( Fig. 1 **b** ). Conservative treatments including percutaneous drainage, total parenteral nutrition, and antibiotics failed to improve the condition.

Reopenable clip-over-the-line method for closure of postoperative gastric fistula.Video 1

**Fig. 1:**
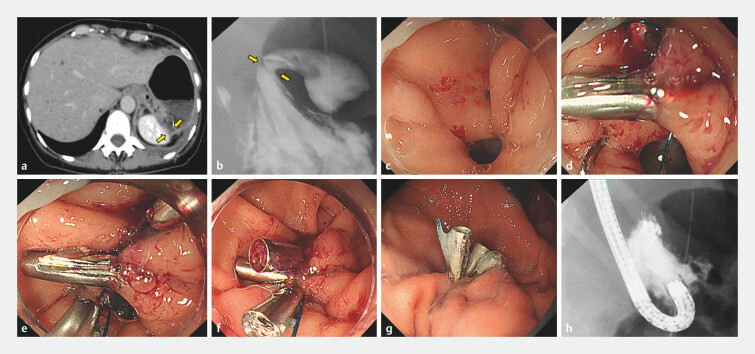
**a** Abdominal CT image reveals a defect in the gastric wall with leakage to the left subdiaphragmatic cavity (arrows). **b** Gastrography revealed the presence of a fistula (arrows). **c** The postoperative pancreatic fistula following a distal pancreatectomy for pancreatic cancer. **d** A clip with a line was first placed at the edge of the fistula. **e** A reopenable clip with a nylon line was applied to the contralateral edge of the fistula. **f** Pulling the nylon line approximated the two clips and achieved complete closure. **g** The fistula was completely closed 7 days after ROLM. **h** The complete closure of the fistula was confirmed by gastrography. Abbreviation: ROLM, reopenable clip-over-the-line method.

On POD 24, endoscopic closure was attempted. An initial attempt with the OTSC system was not pursued, as the rigidity of the surrounding gastric tissue suggested a high risk of failure. We then performed ROLM using a 3–0 monofilament nylon line and seven reopenable clips (SureClip 8 mm; MicroTech), achieving complete closure in 15 minutes ( Fig. 1 **c–g** ). Fluoroscopy confirmed the absence of leakage into the abdominal cavity ( Fig. 1 **h** ).

This case suggests that ROLM is not only effective for mucosal defects but also feasible for full-thickness gastric fistula closure. ROLM may offer a valuable and less complex alternative when conventional approaches are technically challenging.

Endoscopy_UCTN_Code_TTT_1AO_2AI

## Notes

Endoscopy E-Videos https://eref.thieme.de/e-videos
*E-Videos* is an open access online section of the journal *Endoscopy* , reporting on interesting cases and new techniques in gastroenterological endoscopy. All papers include a high-quality video and are published with a Creative Commons CC-BY license. Endoscopy E-Videos qualify for HINARI discounts and waivers and eligibility is automatically checked during the submission process. We grant 100% waivers to articles whose corresponding authors are based in Group A countries and 50% waivers to those who are based in Group B countries as classified by Research4Life (see: https://www.research4life.org/access/eligibility/ ). This section has its own submission website at https://mc.manuscriptcentral.com/e-videos .
